# Linc00941 regulates esophageal squamous cell carcinoma via functioning as a competing endogenous RNA for miR-877-3p to modulate PMEPA1 expression

**DOI:** 10.18632/aging.203286

**Published:** 2021-07-13

**Authors:** Yan Zhang, Huayun Zhu, Ning Sun, Xiaomei Zhang, Geyu Liang, Jiali Zhu, Lei Xia, Yingying Kou, Jianwei Lu

**Affiliations:** 1 Department of Medical Oncology, Jiangsu Cancer Hospital and Jiangsu Institute of Cancer Research and the Affiliated Cancer Hospital of Nanjing Medical University, Nanjing 210009, China; 2 School of Public Health, Southeast University, Nanjing 210009, China

**Keywords:** ESCC, linc00941, miR-877-3p, PMPEA1, EMT

## Abstract

Esophageal squamous cell carcinoma (ESCC) represents one of the most common malignancies and is the fifth leading cause of cancer-related deaths. Long intergenic non-coding RNAs (lincRNAs) have been suggested to be dysregulated in various types of cancers, and a growing number of lincRNAs have been implicated to be functional in the ESCC progression. In this study, we examined the role of linc00941 in the ESCC progression and explored the underlying molecular mechanisms. The bioinformatics analysis identified the up-regulation of linc00941 in the ESCC tissues. Further *in vitro* studies showed that linc00941 was up-regulated in ESCC cell lines. The loss-of-function studies demonstrated that linc00941 knockdown suppressed ESCC cell proliferation, invasion and migration, and also suppressed the *in vivo* tumor growth. Furthermore, bioinformatics prediction along with luciferase reporter assay and RNA immunoprecipitation assay implied that linc00941 acted as a competing endogenous RNA for miR-877-3p, and linc00941 regulated ESCC cell progression via at least targeting miR-877-3p. Subsequently, miR-877-3p targeted prostate transmembrane protein, androgen induced 1 (PMEPA1) 3’ untranslated region and repressed PMEPA1 expression in ESCC cells; overexpression of PMEPA1 attenuated the inhibitory effects of linc00941 knockdown on the ESCC cell progression. Linc00941 knockdown suppressed epithelial-mesenchymal transition (EMT) via targeting miR-877-3p/PMEPA1 axis in ESCC cells. In conclusion, our results indicated the oncogenic role of linc00941 in ESCC, and knockdown of linc00941 suppressed ESCC cell proliferation, invasion, migration and EMT via interacting with miR-877-3p/PMEPA1 axis.

## INTRODUCTION

Esophageal cancer represents a common type of human malignancy and is one of the major causes of cancer-related mortality [1]. Esophageal adenocarcinoma and esophageal squamous cell carcinoma (ESCC) are the major subtypes of esophageal cancer. Based on the epidemiology of cancer statistics, ESCC accounts for more than 80% of the total cases of esophageal cancer in China [1]. Although tremendous progress has been made in clinical aspects for treating ESCC, patients with advanced ESCC usually had a 5-year overall survival rate of less than 30% [2]. As far as we know, the detailed mechanisms of ESCC carcinogenesis have not been fully revealed due to the complex signalling processes involved in the pathophysiology of ESCC [3]. In this regard, it is urgent to uncover potential mechanisms underlying ESCC progression, which may subsequently help with identifying novel theranostic targets for treating ESCC.

Long intergenic ncRNAs (lincRNAs) are transcribed from intergenic regions of protein-coding genes, and belong to non-coding RNA family of > 200 nucleotides in length [4]. Up to date, thousands of lincRNAs are being characterized in the human genome and are associated with various pathological and physiological responses [4]. Mechanistically, lincRNAs can recruit protein complexes and create a locus-specific address to subsequently modulate gene transcription [4]. In addition, lincRNAs are proposed to act as competing endogenous RNAs (ceRNAs) for microRNAs (miRNAs), which consequently regulate the targeted genes [5]. There is growing evidence implying that disruption of the equilibrium of competing endogenous RNA (ceRNA) networks participates in the pathophysiology of various diseases, particularly cancer progression [5]. For examples, LincRNA-regulator of reprogramming acted as ceRNA for miR-145 and promoted breast cancer invasion and metastasis via targeting Mucin 1 [6]. LINC00511 regulated breast cancer tumorigenesis and stemness via targeting miR-185-p3/E2F1/Nanog axis [7]. Mu et al., showed that lincRNA small nucleolar RNA host gene 1 could act on EZH2/miR-154-5p signaling to subsequently regulate colorectal cancer cell progression [8]. In the ESCC, several lincRNAs such as FMR1 antisense RNA 1, TTN antisense RNA 1, ZNFX1 antisense RNA 1 and cancer susceptibility candidate 9 were found to regulate ESCC progression [9–12]. As there are numerous lincRNAs in the human genome, identification of functional lincRNAs in ESCC is still challenging. Owing to the development of high-throughput technologies, RNA sequencing and microarrays have enabled us to identify novel lincRNAs in a more efficient manner [13–17].

Here, we analysed the common differentially expressed lincRNAs in the GSE29986, GSE32424 and GSE130078 datasets using the bioinformatics analysis. Linc00941in the ESCC tissues and cell lines was demonstrated to be up-regulated. In this regard, we further performed *in vitro* functional studies to examine the underlying role of linc00941 in regulating ESCC progression. The present study may bring novel insights into the role of linc00941 in ESCC pathophysiology.

## RESULTS

### Linc00941 exhibited high expression in ESCC tissues

**Figure 1:**
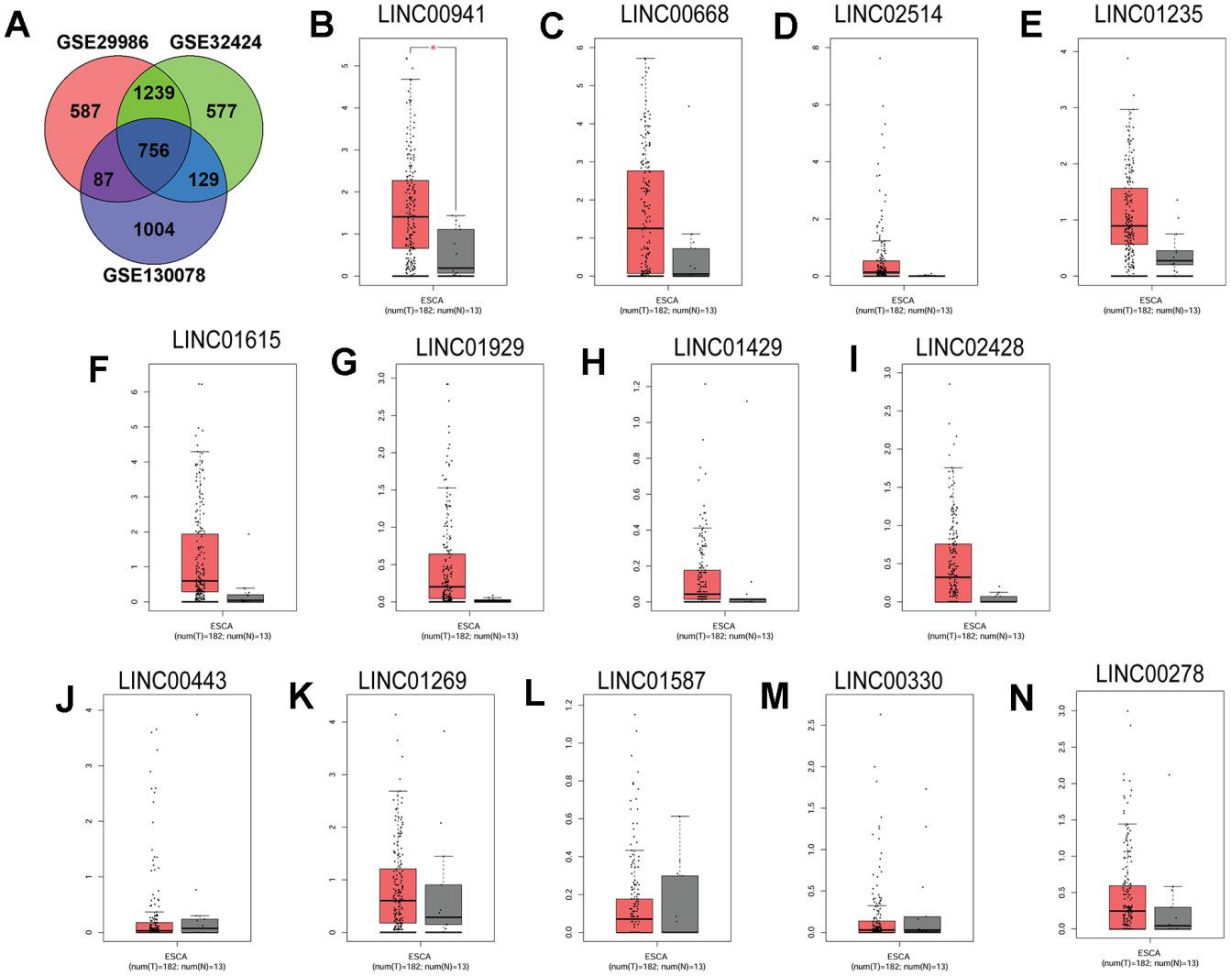
**Linc00941 was up-regulated in the ESCC tissues.** (**A**) Venn diagram of the common DEGs among three GEO datasets (GSE29986, GSE32424 and GSE130078). The expression of (**B**) LINC00941, (**C**) LINC00668, (**D**) LINC02514, (**E**) LINC01235, (**F**) LINC01615, (**G**) LINC01929, (**H**) LINC01429, (**I**) LINC02428, (**J**) LINC00443, (**K**) LINC01269, (**L**) LINC01587, (**M**) LINC00330 and (**N**) LINC00278 in ESCC tissues (n = 182) and normal oesophageal tissues (n = 13). The expression of lincRNAs was extracted from TGCA database. *P<0.05.

Firstly, differentially expressed genes (DEGs) from three GSE29986, GSE32424 and GSE130078 datasets were analysed. Based on bioinformatics analysis, 756 common DEGs were detected among the three datasets. Among these DEGs, a total of 13 lincRNAs were found (Figure 1A). To confirm the underlying roles of these lincRNAs in ESCC progression, these lincRNAs expression in the ESCC tissues and normal esophageal tissues were validated by using the TGCA database. As illustrated in Figure 1B, linc00941 expression was significantly up-regulated in the ESCC tissues compared to that in normal esophageal tissues; whereas no significant difference was detected between the ESCC and normal group in the expression levels of other lincRNAs (linc00668, linc02514, linc01235, linc01615, linc01929, linc01429, linc02428, linc00443, linc01269, linc01587, linc00330 and linc00278) (Figure 1C–1N). Collectively, these preliminary results suggest that linc00941 may involve in the ESCC progression.

### Linc00941 knockdown attenuated ESCC cell proliferation, invasion and migration

**Figure 2:**
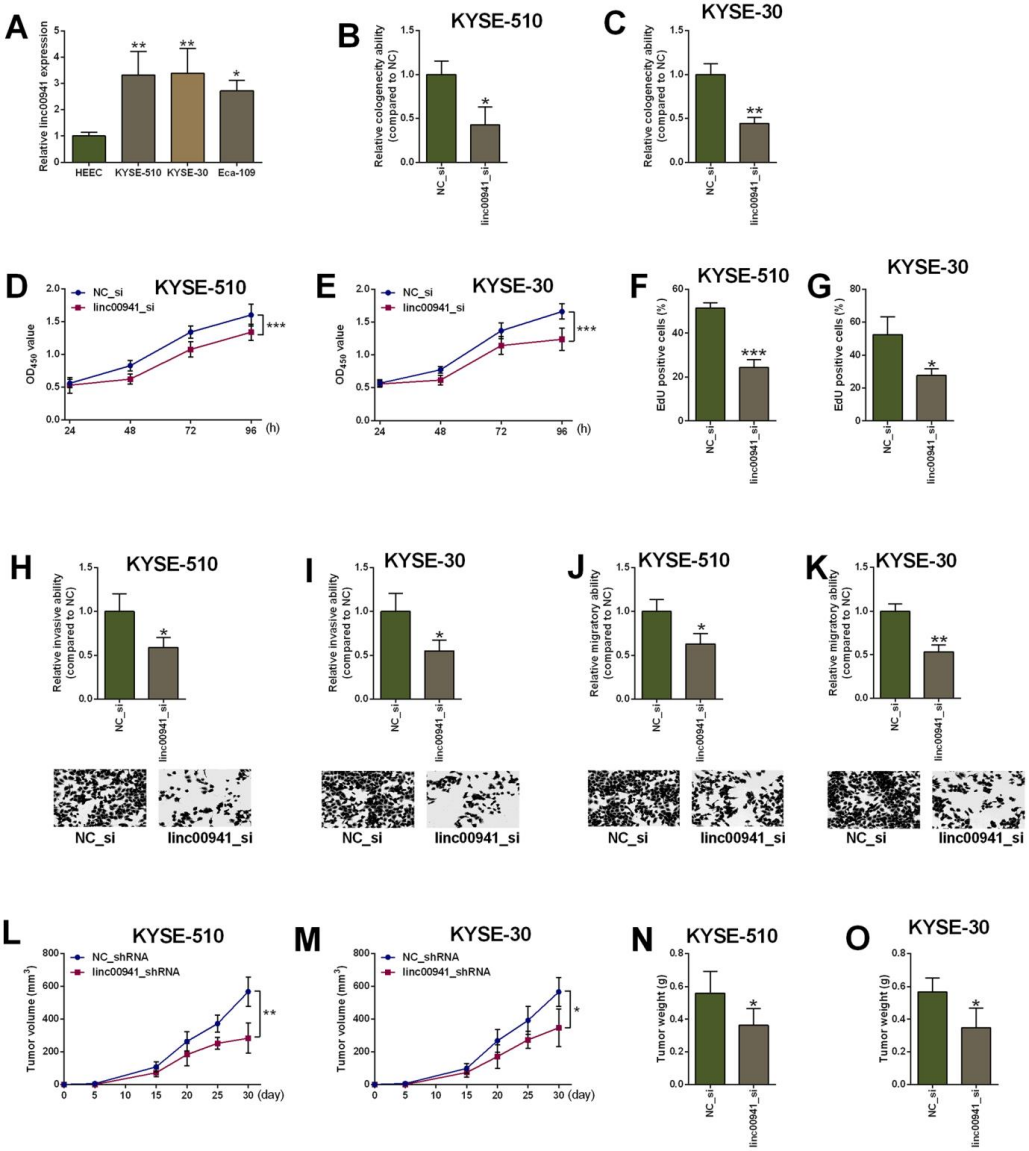
**Knockdown of linc00941 suppressed ESCC cell proliferation, invasion and migration.** (**A**) Linc00941 was up-regulated in ESCC cell lines as determined by qRT-PCR. (**B**, **C**) Linc00941 siRNA transfection attenuated the clonogenicity ability of KYSE-510 and KYSE-30 cells. (**D**, **E**) Knockdown of linc00941 suppressed the proliferative ability of KYSE-510 and KYSE-30 cells as determined by CCK-8 assay. (**F**, **G**) Knockdown of linc00941 attenuated the KYSE-510 and KYSE-30 cell proliferation as determined by EdU assay. (**H**, **I**) Linc00941 siRNA transfection reduced the number of invaded KYSE-510 and KYSE-30 cells as determined by Transwell invasion assay. (**J**, **K**) Linc00941 siRNA transfection retarded wound closure of KYSE-510 and KYSE-30 cells as determined by Wound healing assay. (**L**, **M**) Linc00941 knockdown suppressed the tumor growth of the KYSE-510 and KYSE-30 cells. (**N**, **O**) The tumor weight dissected from the nude mice in different groups was determined. N = 3-6. *P<0.05, **P<0.01 and ***P<0.001 compared between different treatment groups.

The *in vitro* studies were further undertaken to examine the actions of linc00941 in ESCC cell proliferation, invasion and migration. The colony formation, CCK-8 and EdU assays were employed to determine regulatory role of linc00941 in ESCC cell (KYSE-510 and KYSE-30 cells) proliferation. In consistent with the findings in clinical samples, linc00941 expression was up-regulated in the ESCC cells (KYSE-510, KYSE-30 and Eca-109 cells) when compared to that in the HEEC cells (Figure 2A). The knockdown of linc00941 was performed by transfecting KYSE-510 and KYSE-30 cells with linc00941 siRNAs (linc00941_si and linc00941_si#1), and transfection with these two siRNAs significantly down-regulated linc00941 expression when compared to NC_si group (Supplementary Figure 1A, 1B). Moreover, linc00941 knockdown reduced the colony number of KYSE-510 and KYSE-30 cells when compared to NC_si group (Figure 2B, 2C). The CCK-8 assay results showed that linc00941_si transfection significantly repressed the cell proliferation of KYSE-510 and KYSE-30 cells (Figure 2D, 2E). The EdU assay showed that linc00941 knockdown significantly reduced the number of EdU-positive KYSE-510 and KYSE-30 cells when compared to NC_si transfection (Figure 2F, 2G). Collectively, linc00941 knockdown exerted suppressive actions in both cell lines. In addition, the transwell invasion assay results showed that KYSE-510 and KYSE-30 cells with linc00941 siRNA transfection showed decreased invasive ability when compared to NC_si transfection (Figure 2H, 2I). Consistently, linc00941 knockdown remarkably impaired the wound healing capacity of KYSE-510 and KYSE-30 cells when compared to NC_si group (Figure 2J, 2K). The *in vivo* studies deciphered that linc00941 knockdown remarkably attenuated the tumor progression of the KYSE-510 and KYSE-30 cells in the nude mice (Figure 2L, 2M). Tumor weight from the linc00941_shRNA group was lower than that from the NC_shRNA group (Figure 2N, 2O).

### Linc00941 targeted miR-877-3p and attenuated its expression

**Figure 3:**
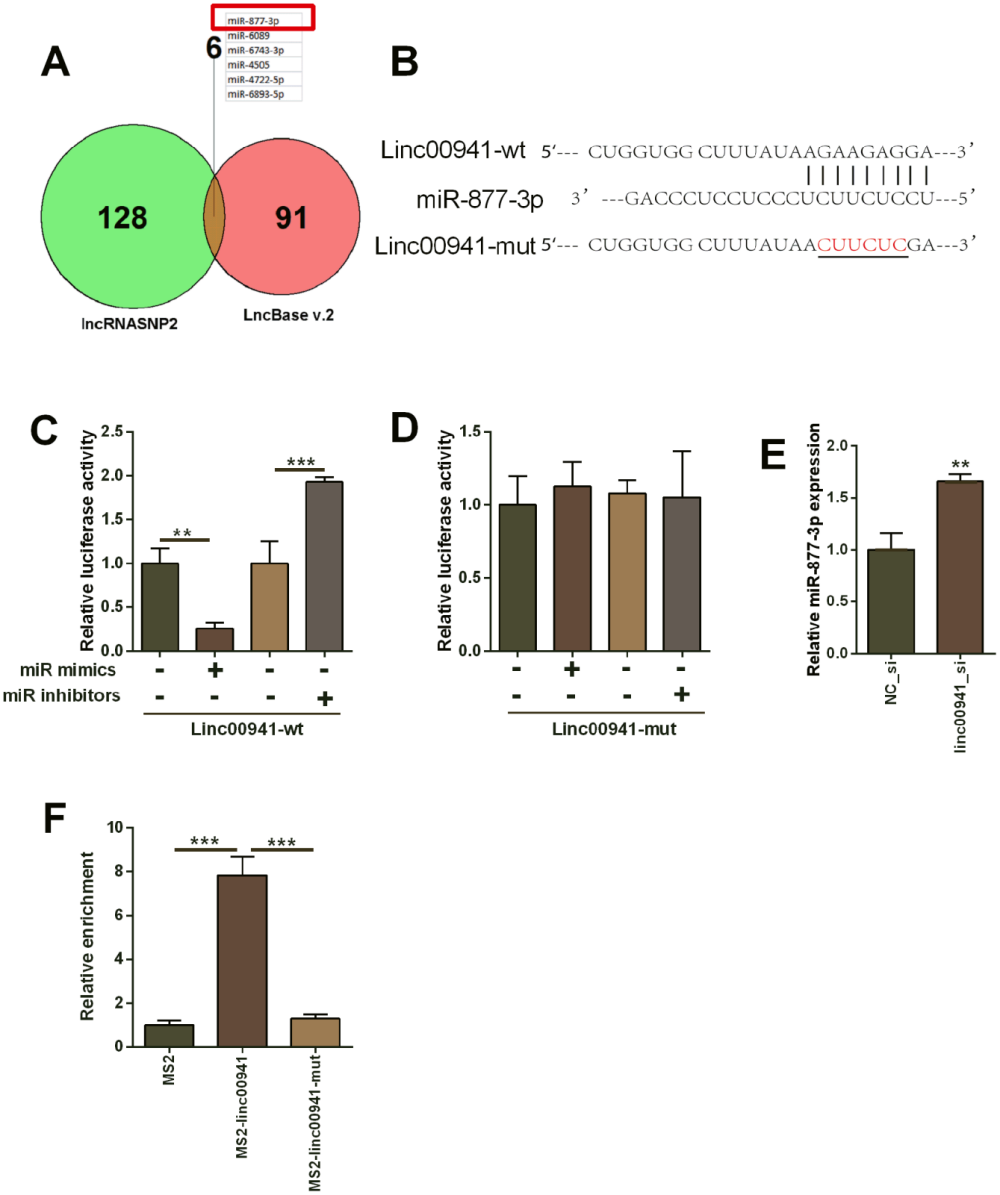
**Linc00941 targeted miR-877-3p and repressed the expression of miR-877-3p.** (**A**) The common miRNAs that could be targeted by linc00941 as predicted by the lncRNASNP2 and LncBase V.2 online predicting tools. (**B**) The predicted binding sites between linc00941 and miR-877-3p. The red letters showed the mutant sites in linc00941 segment used for constructing the mutant luciferase reporter vector. (**C**, **D**) Luciferase activity of linc00941-wt reporter vectors and linc00941-mut reporter vectors in KYSE-510 cells after transfecting with different miRNAs. (**E**) Knockdown of linc00941 up-regulated miR-877-3p expression in KYSE-510 cells as determined by qRT-PCR. (**F**) MS2-RIP assay followed by qRT-PCR to determine miR-877-3p that endogenously associated with linc00941 in KYSE-510 cells. N = 3. **P<0.01 and ***P<0.001 compared between different treatment groups.

As lincRNA could act as ceRNAs for miRNAs, the potential miRNAs that could be targeted by linc00941 were predicted using the lncRNASNP2 and LncBase v.2 online prediction tool. A total of 6 miRNAs (miR-877-3p, miR-6089, miR-6743-3p, miR-4505, miR-4722-5p and miR-6893-5p) were predicted in both databases (Figure 3A). As miR-877-3p had the highest score for binding with linc00941, further investigation was focused on miR-877-3p. The complementary sequences between linc00941 and miR-877-3p were presented in Figure 3B. The mutant sites in the linc00941 were generated using the site-directed mutagenesis (Figure 3B). Moreover, miR-877-3p mimics transfection into KYSE-510 (Supplementary Figure 1C) suppressed the luciferase activity of linc00941-wt reporter vector but not linc00941-mut reporter vector when compared to mimics NC transfection (Figure 3C, 3D). On the other hand, miR-877-3p inhibitors transfection (Supplementary Figure 1C) increased luciferase activity of linc00941-wt reporter vector but not linc00941-mut reporter vector when compared to inhibitors NC transfection (Figure 3C, 3D). Furthermore, linc00941 siRNA transfection into KYSE-510 cells significantly up-regulated miR-877-3p expression when compared to NC_si transfection (Figure 3E). The MS2-RIP assay revealed that miR-877-3p was enriched in the MS2-linc00941-wt but not in the MS2-linc00941-mut (Figure 3F), suggesting the physical interaction between linc00941 and miR-877-3p.

### Inhibition of miR-877-3p attenuated suppressive effects of linc00941 knockdown on ESCC cell progression

**Figure 4:**
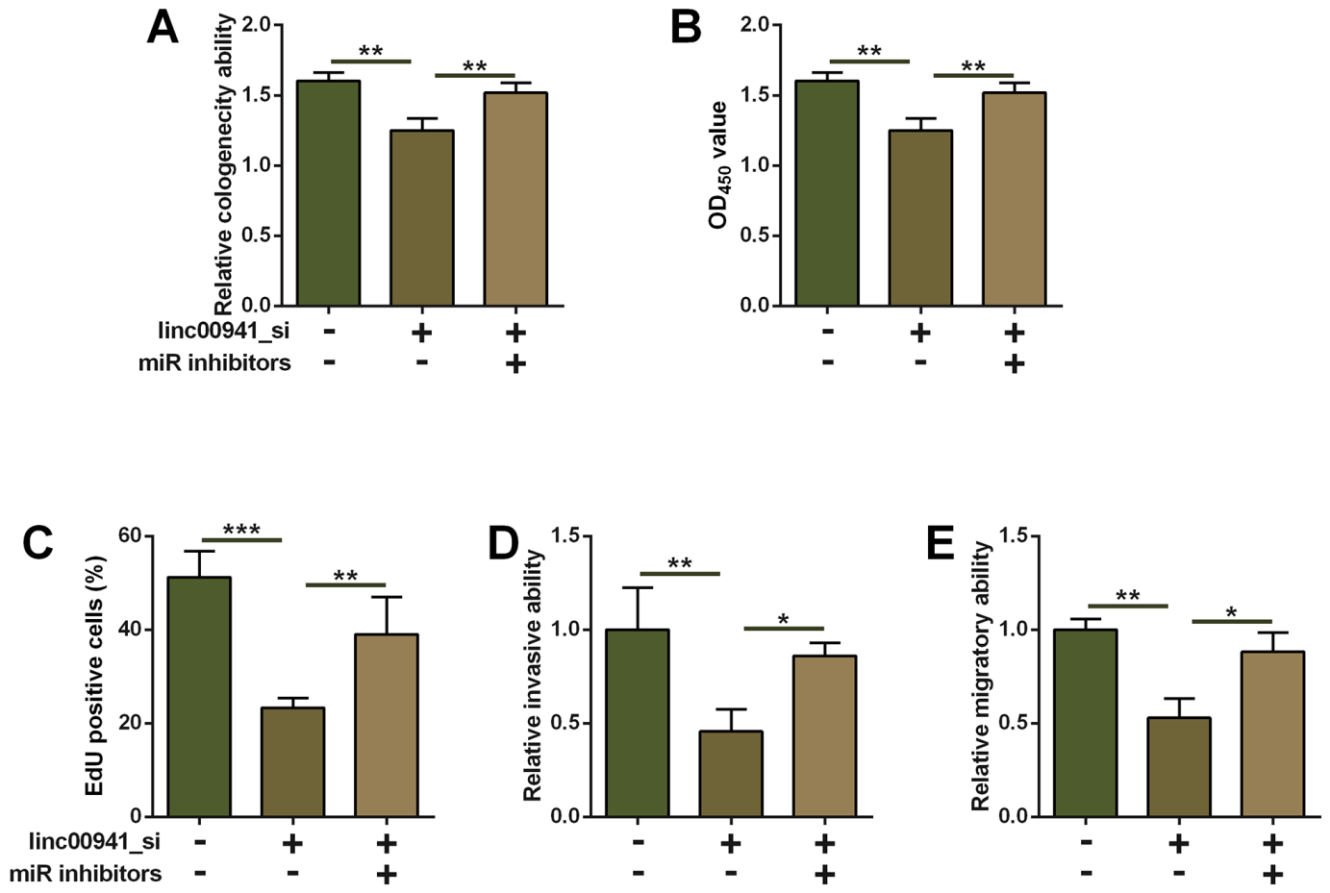
**Inhibition of miR-877-3p attenuated the suppressive effects of linc00941 knockdown on ESCC cell progression.** (**A**) Colony formation assay, (**B**) CCK-8 assay and (**C**) EdU assay determined the KYSE-510 cell proliferation. KYSE-510 cells were co-transfected with NC_si + inhibitors NC, linc00941_si + inhibitors NC, or linc00941_si + miR inhibitors. (**D**) Transwell invasion assay and (**E**) wound healing assay determined the KYSE-510 cell invasion and migration. KYSE-510 cells were co-transfected with NC_si + inhibitors NC, linc00941_si + inhibitors NC, or linc00941_si + miR inhibitors. N = 3. **P<0.01 and ***P<0.001 compared between different treatment groups.

The underlying role of miR-877-3p in linc00941-mediated effects on ESCC cell progression was evaluated by the rescue experiments. As shown in Figure 4A, miR-877-3p inhibitors transfection increased the number of colonies in the linc00941_si-transfected KYSE-510 cells when compared to that co-transfected by linc00941_si and inhibitors NC (Figure 4A). KYSE-510 cells co-transfected by linc00941_si and miR-877-3p inhibitors showed enhanced cell proliferative ability when compared to that transfected with linc00941_si and inhibitors NC (Figure 4B, 4C). Consistently, miR-877-3p inhibitors transfection partially antagonized the suppressive effects of linc00641_si on the KYSE-510 cell invasion and migration (Figure 4D, 4E). Taken together, the suppressive actions of linc00941 knockdown in ESCC cell proliferation, invasion and migration were mediated at least via miR-877-3p.

### MiR-877-3p targeted *PMEPA1* and repressed the expression of *PMEPA1*

**Figure 5:**
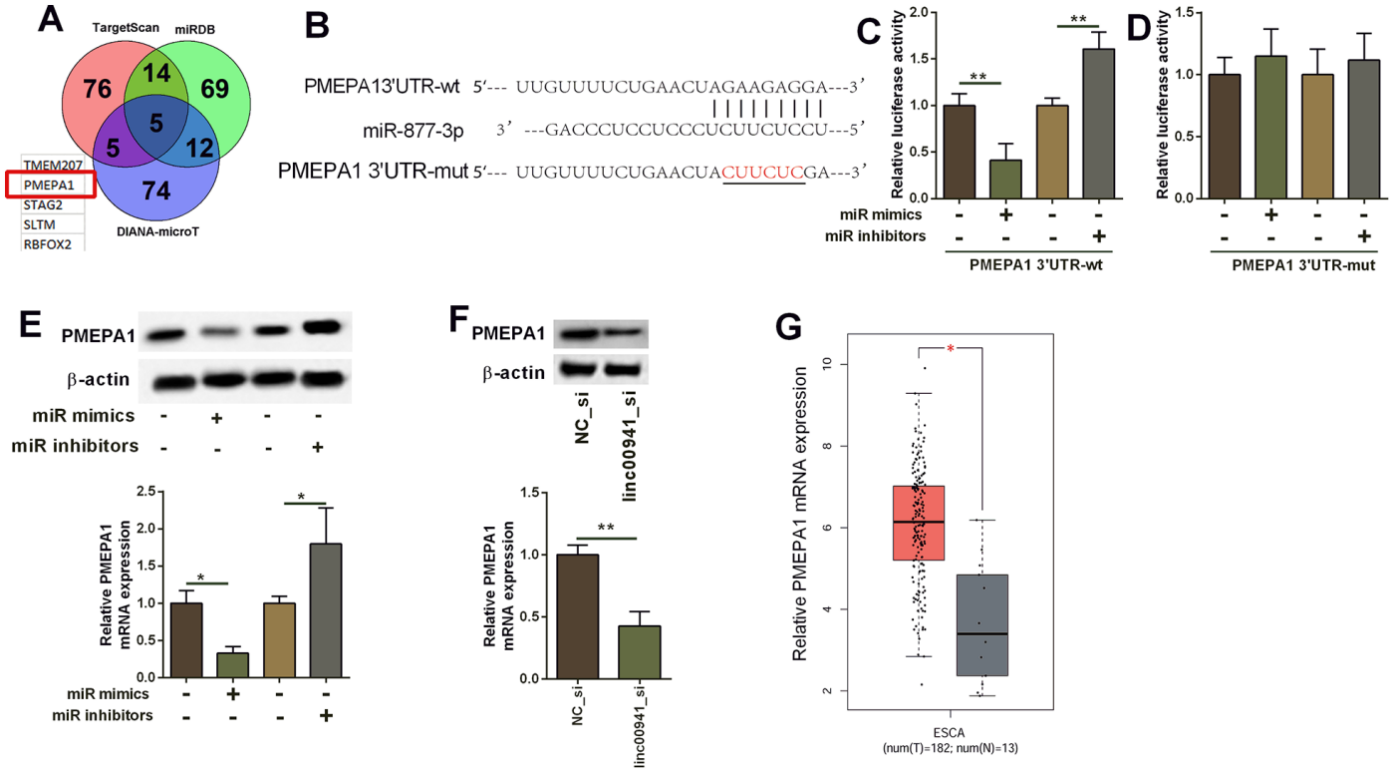
**MiR-877-3p targeted PMEPA1 and repressed the expression of PMEPA1.** (**A**) The common genes that could be targeted by miR-877-3p as predicted by the TargetScan, miRDB and DIANA-microT online predicting tools. (**B**) The predicted binding sites between PMEPA1 3’UTR and miR-877-3p. The red letters showed the mutant sites in PMEPA1 3’UTR used for constructing the mutant luciferase reporter vector. (**C**, **D**) Luciferase activity of PMEPA1 3’UTR-wt reporter vectors and PMEPA1 3’UTR-mut reporter vectors in KYSE-510 cells after transfecting with different miRNAs. (**E**) Western blot assay and qRT-PCR determined the protein and mRNA expression levels of PMEPA1 in KYSE-510 cells. KYSE-510 cells were transfected with mimics NC, miR mimics, inhibitors NC or miR inhibitors. (**F**) Knockdown of linc00941 down-regulated PMEPA1 expression in KYSE-510 cells as determined by western blot and qRT-PCR assays. (**G**) The expression of PMEPA1 in ESCC tissues (n = 182) and normal oesophageal tissues (n = 13); The expression of lincRNAs was extracted from TGCA database. *P<0.05 and **P<0.01 compared between different treatment groups.

The targets of miR-877-3p were analysed by TargetScan, miRDB and DIANA-microT online prediction tool. Based on the predicted results, five common genes were predicted by the three databases, and the targets include *TMEM207*, *PMEPA1*, *STGA2*, *SLTM* and *RBFOX2* (Figure 5A). Furthermore, we focused on *PMEPA1* for further investigation. As shown in Figure 5B, the complementary sequences between miR-877-3p and PMEPA1 3’UTR have 8 complementary nucleotides. Mutant *PMEPA1* 3’UTR was generated by site-directed mutagenesis, and was subsequently used for constructing the *PMEPA1* 3’UTR-mut luciferase reporter vector (Figure 5B). MiR-877-3p mimics transfection into KYSE-510 suppressed the luciferase activity of PMEPA1 3’UTR-wt reporter vector but not *PMEPA1* 3’UTR-mut reporter vector when compared to mimics NC transfection (Figure 5C, 5D). On the other hand, miR-877-3p inhibitors transfection increased the luciferase activity of the *PMEPA1* 3’UTR-wt reporter vector but not *PMEPA1* 3’UTR-mut reporter vector when compared to inhibitors NC transfection (Figure 5C, 5D). MiR-877-3p overexpression suppressed *PMEPA1* mRNA and protein expression level in KYSE-510 cells; whereas miR-877-3p knockdown promoted *PMEPA1* expression in KYSE-510 cells (Figure 5E). In addition, linc00941 knockdown significantly down-regulated *PMEPA1* expression (Figure 5F). The bioinformatics analysis using TGCA database uncovered that *PMEPA1* expression level was obviously higher in the ESCC tissues than that in normal oesophageal tissues (Figure 5G).

### Overexpression of *PMEPA1* attenuated the suppressive effects of linc00941 knockdown on ESCC cell progression

**Figure 6:**
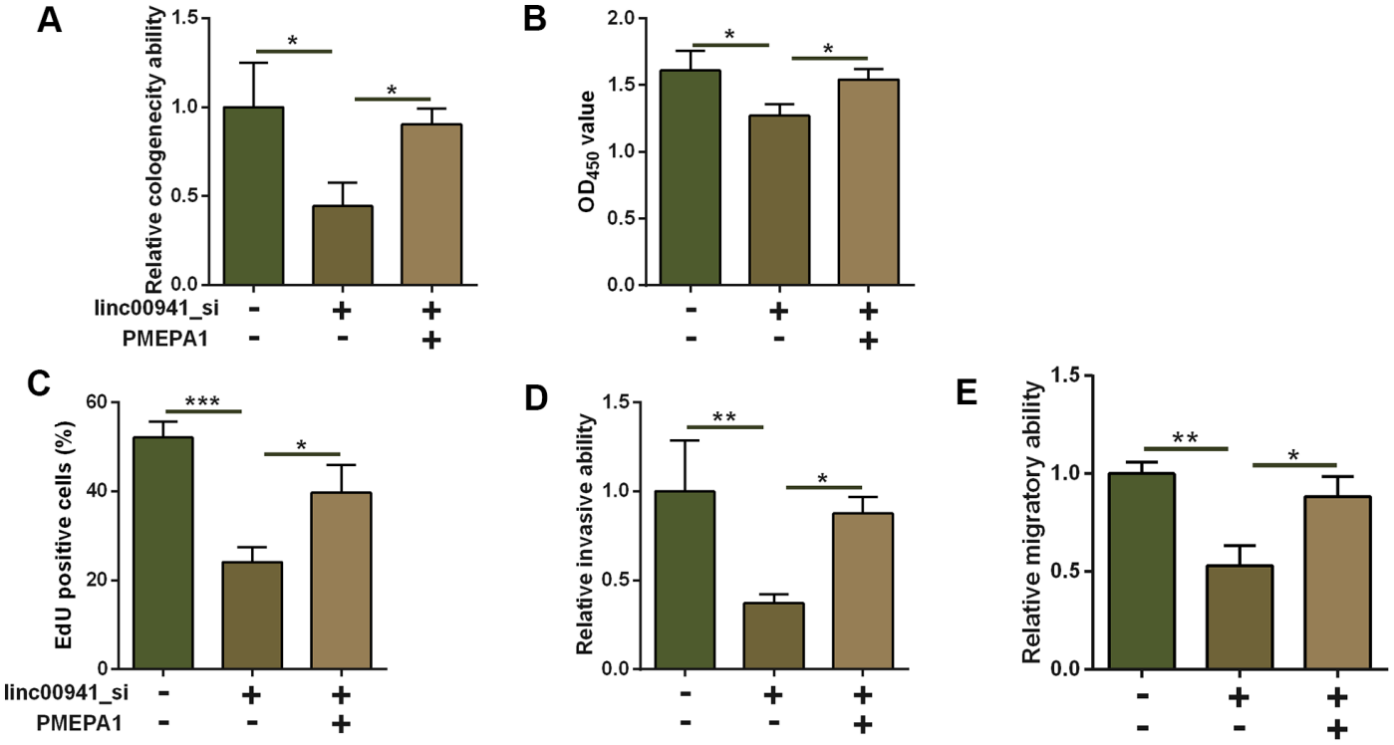
**Overexpression of PMEPA1 attenuated the suppressive effects of linc00941 knockdown on ESCC cell progression.** (**A**) Colony formation assay, (**B**) CCK-8 assay and (**C**) EdU assay determined the KYSE-510 cell proliferation. KYSE-510 cells were co-transfected with NC_si + pcDNA, linc00941_si + pcDNA, or linc00941_si + PMEPA1. (**D**) Transwell invasion assay and (**E**) wound healing assay determined the KYSE-510 cell invasion and migration. KYSE-510 cells were co-transfected with NC_si + pcDNA, linc00941_si + pcDNA, or linc00941_si + PMEPA1. N = 3. *P<0.05, **P<0.01 and ***P<0.001 compared between different treatment groups.

As PMEPA1 was up-regulated in the ESCC tissues, *PMEPA1* may act as an oncogene in ESCC. In this regard, we examined if *PMEPA1* overexpression could attenuate the tumor-suppressive effects of linc00941 siRNA on the KEYSE-510 cells. *PMEPA1* overexpression was performed by transfecting pcDNA-PMEPA1 into KYSE-510 cells (Supplementary Figure 1D). Based on the results from colony formation assay, CCK-8 assay as well as EdU assay, *PMEPA1* overexpression enhanced the proliferative capacity of KYSE-510 cells with linc00941 knockdown when compared to linc00941_si-trasfected KYSE-510 cells with pcDNA transfection (Figure 6A–6C). Moreover, the overexpression of *PMEPA1* also attenuated the suppressive effects of linc00941 knockdown on the invasive and migratory capacity of KYSE-510 cells (Figure 6D, 6E).

### Knockdown of linc00941 suppressed EMT via miR-877-3p/PMEPA1 axis in KYSE-510 cells

**Figure 7:**
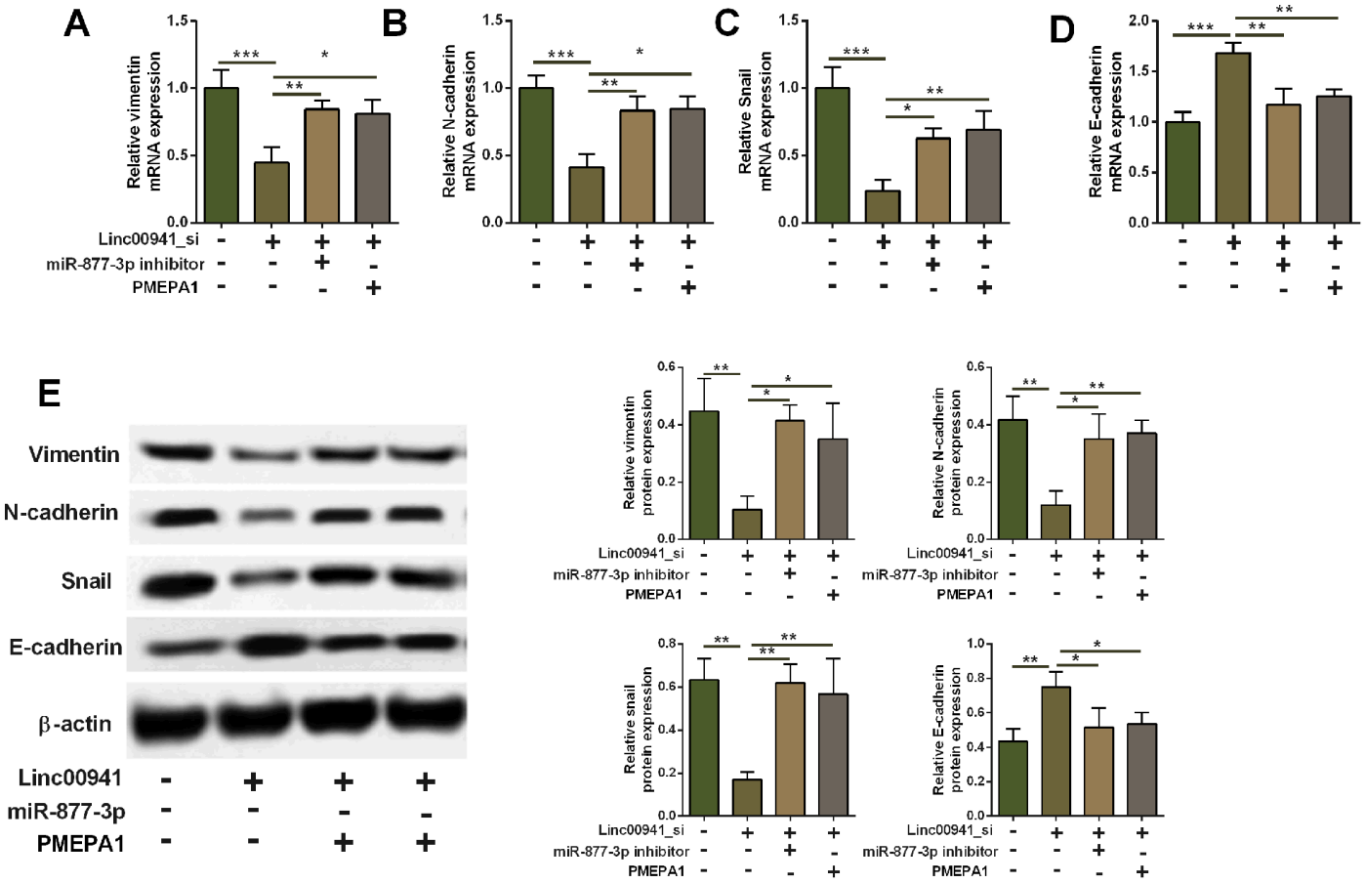
**Knockdown of linc00941 suppressed EMT via miR-877-3p/PMEPA1 axis in KYSE-510 cells.** (**A**–**D**) qRT-PCR determined the mRNA expression of vimentin, N-cadherin, Snail and E-cadherin in KYSE-510 cells transfected with linc00941 siRNA, miR-877-3p inhibitors, pcDNA-PMEPA1 or the respective controls. (**E**) Western blot assay determined the protein expression of vimentin, N-cadherin, Snail and E-cadherin in KYSE-510 cells transfected with linc00941 siRNA, miR-877-3p inhibitors, pcDNA-PMEPA1 or the respective controls. N = 3. *P<0.05, **P<0.01 and ***P<0.001 compared between different treatment groups.

As *PMEPA1* has been shown to regulate EMT in the cancer progression, the action of linc00941/miR-877-3p/PMEPA1 axis in regulating EMT in KYSE-510 cells was further explored. Firstly, we performed qRT-PCR assay to determine vimentin, N-cadherin, Snail and E-cadherin mRNA expression. Knockdown of linc00941 down-regulated vimentin, N-cadherin and snail expression, but E-cadherin expression, suggesting that linc00941 knockdown exerts inhibitory effects on the EMT in KYSE-510 cells. In addition, miR-877-3p inhibition and *PMEPA1* overexpression both attenuated the suppressive effects of linc00941 knockdown on the EMT in KYSE-510 cells (Figure 7A–7D). Furthermore, the western blot results consistently showed that linc00941 knockdown repressed N-cadherin, vimentin and snail protein levels, while increased E-cadherin protein level, which was attenuated by miR-877-3p inhibition and *PMEPA1* overexpression in KYSE-510 cells (Figure 7E). Taken together, these results implied that linc00941 knockdown suppressed EMT via miR-877-3p/PMEPA1 axis in KYSE-510 cells.

## DISCUSSION

LincRNAs have been suggested to be dysregulated in various types of cancers. Up to date, lots of lincRNAs have been implicated to be functional in ESCC progression [18]. In this study, we performed the bioinformatics analysis and identified the up-regelation of linc00941 in the ESCC tissues. Further *in vitro* studies consistently revealed that linc00941 was highly expressed in the ESCC cell lines. The loss-of-function studies demonstrated that linc00941 knockdown impaired ESCC cell proliferation, invasion and migration, and also repressed *in vivo* tumor growth. Furthermore, bioinformatics prediction along with luciferase reporter assay and MS2-RIP assay implied that linc00941 acted as a ceRNA for miR-877-3p, and linc00941 regulated ESCC cell progression via at least targeting miR-877-3p. Subsequently, miR-877-3p targeted *PMEPA1* 3’UTR and repressed *PMEPA1* expression in ESCC cells; overexpression of *PMEPA1* attenuated inhibitory effects of linc00941 knockdown on the ESCC cell progression. Moreover, we also demonstrated that linc00941 knockdown suppressed EMT via targeting miR-877-3p/PMEPA1 axis in ESCC cells. Taken together, our data suggested the oncogenic role of linc00941 in ESCC, and linc00941 silence impaired ESCC cell proliferation, invasion, migration and EMT via interacting with miR-877-3p/PMEPA1 axis.

Linc00941 expression has been detected in several types of cancers. Zhang et al., proved that linc00941 acted as a candidate prognostic biomarker in lung cancer [19], and alterations in linc00941 copy number were correlated with lung cancer patients survival status [20]. Luo et al., demonstrated that linc00941 was highly expressed in gastric cancer and correlated with invasion depth, lymphatic metastasis, and the TNM stage of gastric cancer patients [21], and linc00941 promoted gastric carcinoma proliferation and metastasis [22]. In addition, linc00941 was found to be up-regulated in the colon cancer cells [23]. Linc00941 was found to be a novel transforming growth factor beta (TGF-beta) target by recruiting cadherin 6, which subsequently enhanced papillary thyroid carcinoma metastasis [24]. Hu et al., performed the integrated bioinformatics analysis and found that linc00941 was not only an optimal diagnostic lincRNAs biomarkers, but also was a prognostic biomarker in head and neck squamous cell carcinoma [25]. Our data revealed that linc00941 was highly expressed in the ESCC tissues and cell lines, suggesting that linc00941 may be oncogenic in ESCC. Further functional studies showed that linc00941 knockdown impaired ESCC cell progression, which indicated that linc00941 exerted oncogenic actions in the ESCC cells.

The ceRNA hypothesis for lincRNA has been proven by several lines of cancer studies. In this study, we performed the bioinformatics analysis along with functional assays, and showed that miR-877-3p was targeted by linc00941, and linc00941 functioned as a ceRNA for miR-877-3p. Biological functions of miR-877-3p have been elucidated in various studies. Liang et al., showed that miR-877-3p regulated interleukin-8 and interleukin-1 beta production in mesangial cells in nephropathy patients [26]. In the cancer studies, miR-877-3p was found to inhibit bladder cancer cell proliferation and act as a tumor suppressor [27]. In gastric cancer, miR-877-3p exhibited low expression in gastric cancer tissues, and miR-877-3p suppressed gastric cancer metastasis via targeting VEGFA [28]. In this study, we carried out the functional rescue assays, and found that miR-877-3p inhibition attenuated the suppressive actions of linc00941 knockdown in the ESCC cell proliferation and metastasis, suggesting that linc00941 siRNA exerts tumor suppressive effects on ESCC at least via miR-877-3p.

The further bioinformatics analysis using different predicting tools along with luciferase reporter assay showed that *PMEPA1* 3’UTR was targeted by miR-877-3p. *PMEPA1* gene is commonly amplified in several types of solid tumors. Liu et al*.,* showed that *PMEPA1* could attenuate the Smad3/4–c-Myc–p21 signalling thus to modulate prostate cancer cell proliferation [29]. *PMEPA1* could inhibit canonical Smad signalling by reducing phosphatase and tensin homolog in breast cancer [30]. Knockdown of *PMEPA1* impaired lung metastasis and tumor growth of MDA-MB-231 cells [31]. In the colorectal cancer, *PMEPA1* promoted EMT via targeting the non-canonical TGF-beta signalling [32]. In this study, *PMEPA1* was up-regulated in ESCC tissues, and overexpression of *PMEPA1* attenuated the suppressive actions of linc00941 knockdown in ESCC cell proliferation and metastasis. As EMT is a critical process in cancer cell migration and distal metastasis and PMEPA1 is an important regulator for EMT, we further examined if linc00941/miR-877-3p/PMEPA1 axis regulates EMT in ESCC cells. Linc00941 silencing attenuated EMT in ESCC cells, which was attenuated by miR-877-3p inhibition and *PMEPA1* overexpression. Taken together, our data implied that linc00941 regulated ESCC cell proliferation, metastasis and EMT via targeting miR-877-3p/PMEPA1 axis.

We have not performed the clinical studies regarding the role of linc00941/miR-877-3p/PMEPA1 axis in ESCC progression, which is a drawback. By using the bioinformatics analysis, high expression of miR-877-3p predicted better overall survival of patients with ESCC (Supplementary Figure 2B); while the expression levels between linc00941 and PMEPA1 were not significantly correlated with the overall survival of patients with ESCC (Supplementary Figure 2A, 2C), which may be caused by the small sample size in the database. Future studies are warranted to decipher the clinical significance of linc00941/miR-877-3p/PMEPA1 axis in ESCC progression. Though the role of linc00941 has been reported in other cancers, we reported the role of linc00941 in ESCC and also demonstrated the importance of the novel linc00941/miR-877-3p/PMEPA1 axis in ESCC progression. However, as linc0094 may have other downstream targets, we may consider explore other signalling pathways mediated by linc00941 in ESCC in the future studies. In the *in vivo* studies, we only examined the knockdown effects of linc00941 on the *in vivo* tumor growth, and the actions of linc00941 in the *in vivo* tumor metastasis and its related signalling pathways should be determined in the future studies.

## CONCLUSIONS

In conclusion, we for the first time identified a novel linc00941 in ESCC, and linc00941 was highly expressed in ESCC tissues and cell lines. Further mechanistic studies underscored that linc00941 knockdown impaired ESCC cell proliferation, metastasis and EMT via interacting with miR-877-3p/PMEPA1 signalling. However, the detailed molecular mechanisms and clinical significance of linc00941 in ESCC progression still require further examinations.

## MATERIALS AND METHODS

### Collection of microarray data

ESCC gene expression profiling studies were carried out using the GEO database. Three GEO datasets GSE29968, GSE32424 and GSE130078 were downloaded. GSE29968 dataset was based on the GPL10999 Illumina Genome Analyzer IIx and included 3 normal esophageal tissues and 3 ESCC tissues. GSE32424 dataset was based on the GPL10999 Illumina Genome Analyzer IIx and included 5 normal esophageal tissues and 7 ESCC tissues. GSE130078 dataset was based on the GPL11154 Illumina HiSeq 2000 and included 23 normal esophageal tissues and 23 ESCC tissues.

### Data processing and differentially expressed genes (DEGs) extraction

Three GEO datasets GSE29968, GSE32424 and GSE130078 were processed by the EdgeR [33] to identify DEGs between normal esophageal tissues and ESCC tissues. A false discovery rate value < 0.05 and |log fold change (FC)| > 1.5 and were employed as the cut-off criteria for DEGs. Subsequently, overlapping DEGs among GSE29968, GSE32424 and GSE130078 were extracted using iGEAK tool [34].

### Analysis of hub gene expression and survival analysis of targeted genes

Gene Expression Profiling Interactive Analysis (GEPIA) database [35] was employed to visualize the expression of 10 hub genes by using the boxplot. P < 0.05 was regarded to be statistically significant. The overall survival analysis of targeted genes in ESCC patients was analysed by using the Kaplan-Meier Plotter tool (https://kmplot.com/analysis/) [36].

### Cell lines and culture

Human esophageal cancer cell lines KYSE-510, Eca-109 and KYSE-30 and were purchased from Guangzhou Cellcook Cell Biotechnology, Ltd (Guangzhou, China) and were maintained in RPMI 1640 medium (Gibco, Waltham, MA, USA) with 10% fetal bovines serum (FBS; Gibco). Human esophagus epithelial cell line (HEEC) was obtained from the American Type Culture Collection (Manassas, VA, USA) and kept in DMEM medium (Gibco) supplemented with 10% FBS (Gibco). All the cells mere maintained at 37° C in 5% CO_2_ atmosphere.

### Quantitative real-time PCR (qRT-PCR)

RNA was isolated from human esophageal cancer cells/tissues or esophagus epithelial cells by using Trizol reagent (Invitrogen, Waltham, MA, USA). Reverse transcription of 100 ng total RNA was conducted by using PrimeScript 1st strand cDNA Synthesis Kit (Takara, Dalian, China). For miRNA, Bulge-LoopTM miRNA primers (RiboBio, Guangzhou, China) were added during reverse transcription. Real-time PCR was performed on the LightCycler® 480 II Real-time PCR system (Roche, Basel, Switzerland) using SYBR Green I Master (Roche). GAPDH and U6 were served as internal control for mRNA and miRNA, respectively. Relative expression was analysed using the 2^(−ΔΔCt)^ method.

### Oligonucleotides and transfection

PMEPA1-pcDNA3.1 overexpression vector, empty pcDNA3.1 vector, specific siRNAs against linc00941, scrambled siRNA for linc00941, miR-877-3p mimics, mimics NC, miR-877-3p inhibitors and inhibitors NC were designed by RiboBio. The cells were transfected with the oligonucleotides by using Lipofectamine 2000 (Invitrogen; Thermo Fisher Scientific, Inc.) in Opti-MEM medium for different time points. Subsequently, the cells were collected for further studies.

### Colony formation

KYSE-510/30 cells were seeded in 6-well dishes with 1000 cells/well. They were kept in full medium and allowed to grow for two weeks. Subsequently, colonies were fixed, stained with 0.3% crystal violet and quantified under a stereomicroscope. A colony should consist of at least 50 cells.

### Cell counting kit-8 (CCK-8) assay

Cell proliferation was detected by using Cell Counting kit-8 (Dojindo Laboratories, Kumamoto, Japan). Briefly, cells were seeded onto the 96-well plates. After cell transfection, cells were incubated with CCK-8 reagent for 24, 48, 72 and 96 h. Then the cells were kept in incubator for another 2 h at 37° C. Optical density values were evaluated at 450 nm using a microplate reader (Thermo Fisher Scientific, Inc.).

### EdU assay

EdU assay was employed to assess cell proliferative potential using EdU labelling/detection kit (Ribobio). Briefly, after cell transfection, cells were treated with EdU (25 μM). After 12 h, cells were fixed with 4% formaldehyde for 30 min at room temperature followed by permeabilization with 0.5% Triton X-100 for 15 min. After being washed with phosphate buffered saline, cells were reacted with Apollo reaction cocktail for 30 min. followed by counter-staining with Hoechst 33342 for 30 min. The EdU-positive cells were then assessed under a fluorescence microscope.

### Cell migration and invasion assays

Transwell migration and invasion assays were both performed in 24-well transwell inserts with 8 μm pore size membranes (Corning Costar Corp., Corning, NY, USA). For migration assay, 2×10^4^ transfected cells were plated in the upper chamber with culture medium without FBS. For the invasion assay, transfected cells were loaded in the upper Matrigel-coated chamber. In both assays, the lower chambers were filled with corresponding culture medium supplemented with 10% FBS as chemoattractant. After 24 h, cells that migrated or invaded to the bottom chamber were fixed by 100% methanol and stained with 0.3% crystal violet, and then were visualized and quantified under a microscope.

### Dual luciferase reporter assay

The wild-type (wt) or mutant (mut) *PMEPA1* 3′ untranslated region sequence, or wild type linc00941 or mutant linc00941 sequence was cloned into the pmirGLO vector (Promega Corporation, Madison, WI, USA) to form wild type or mutant luciferase reporter vectors. Subsequently, pmirGLO-PMEPA1 3’UTR-wt, pmirGLO-PMEPA1 3’UTR-mut, pmirGLO-linc00941 or pmirGLO-linc00941-mut was co-transfected with miR-877-3p mimics or mimics control, miR-877-3p inhibitors or inhibitors control using Lipofectamine 2000 reagent (Invitrogen, Waltham, MA, USA) in KYSE-510 cells. Luciferase activity was determined 48 h later by using a dual-luciferase reporter assay kit (Promega Corporation).

### RNA immunoprecipitation (RIP) assay

KYSE-510cells were co-transfected pcDNA3.1-MS2, pcDNA3.1-MS2-linc00941, or pcDNA3.1-MS2-linc00941-mut along with pMS2-GFP(Addgene). After 48 h, immunoprecipitation assay was conducted according to the manufacturer’s protocol (Millipore,Burlington, MA, USA). Briefly, KYSE-510 cells with different transfections were lysed in the lysis buffer with protease and RNase inhibitors. RNAs magnetic beads were first incubated with anti-AGO2 antibody (1:1000; Abcam, USA) or negative control anti-IgG (Millipore), and then immune-precipitated RNAs were extracted from RNA-protein complexes. Purified RNA was extracted and proceeded to qRT-PCR analysis.

### Western blot assay

The process of protein samples was described before [37]. The primary antibodies against PMEPA1, vimentin, N-cadherin, Snail, E-cadherin and β-actin was purchased from Cell Signalling Technology (Danvers, MA, USA) and the goat anti-rabbit horseradish peroxidase-conjugated secondary antibody was also from Cell Signalling Technology.

### *In vivo* tumor growth assay

The animal experiments were approved by the Animal Care and Use Committee of The Affiliated Cancer Hospital of Nanjing Medical University. Plasmids for shRNA targeting *linc00941 (linc00941_shRNA)* and scrambled shRNA (NC_shRNA) were obtained from GenePharma Company (Shanghai, China). BALB/c mice (4–6 weeks old) were purchased from Shanghai SLAC Laboratory Animal Co. Ltd. (Shanghai, China). KYSE-510 or KYSE-30 cells (5 × 10^6^ cells/100 μL/mouse) transfected with linc00941_shRNA or NC_shRNA were subcutaneously administered into the left hind flanks of mice. Tumor growth was evaluated by assessing tumor diameters every 5 days for 30 days with a caliper, and tumor volume was calculated using the following formula: V = 1/2×tumor length ×tumor width^2^, where V = tumor volume. The animals were sacrificed on day 30 after implantation, and tumor tissues were collected for analysis.

### Statistical analysis

The data was expressed as mean ± standard deviation and experiments were performed at least in triplicates. GraphPad Prism was used to analyse the data and plot the graph. Student's t-test or one-way ANOVA was adopted to assess the statistical significance of differences between/among groups. P < 0.05 was considered statistically significant.

### Data availability

All the datasets in the manuscript are available.

### Ethics approval and consent to participate

The animal handling procedures were approved by the Animal Welfare Committee of The Affiliated Cancer Hospital of Nanjing Medical University.

## Supplementary Material

Supplementary Figures
