# Supplementary material for: Linc00941 regulates esophageal squamous cell carcinoma via functioning as a competing endogenous RNA for miR-877-3p to modulate PMEPA1 expression
Source: Aging (Albany NY). 2021 Jul 13;13(13):17830–46. doi: 10.18632/aging.203286 (PMC8312468; doi:10.18632/aging.203286)
Supplement: Supplementary Figures [file aging-13-203286-s001.pdf]

SUPPLEMENTARY FIGURES

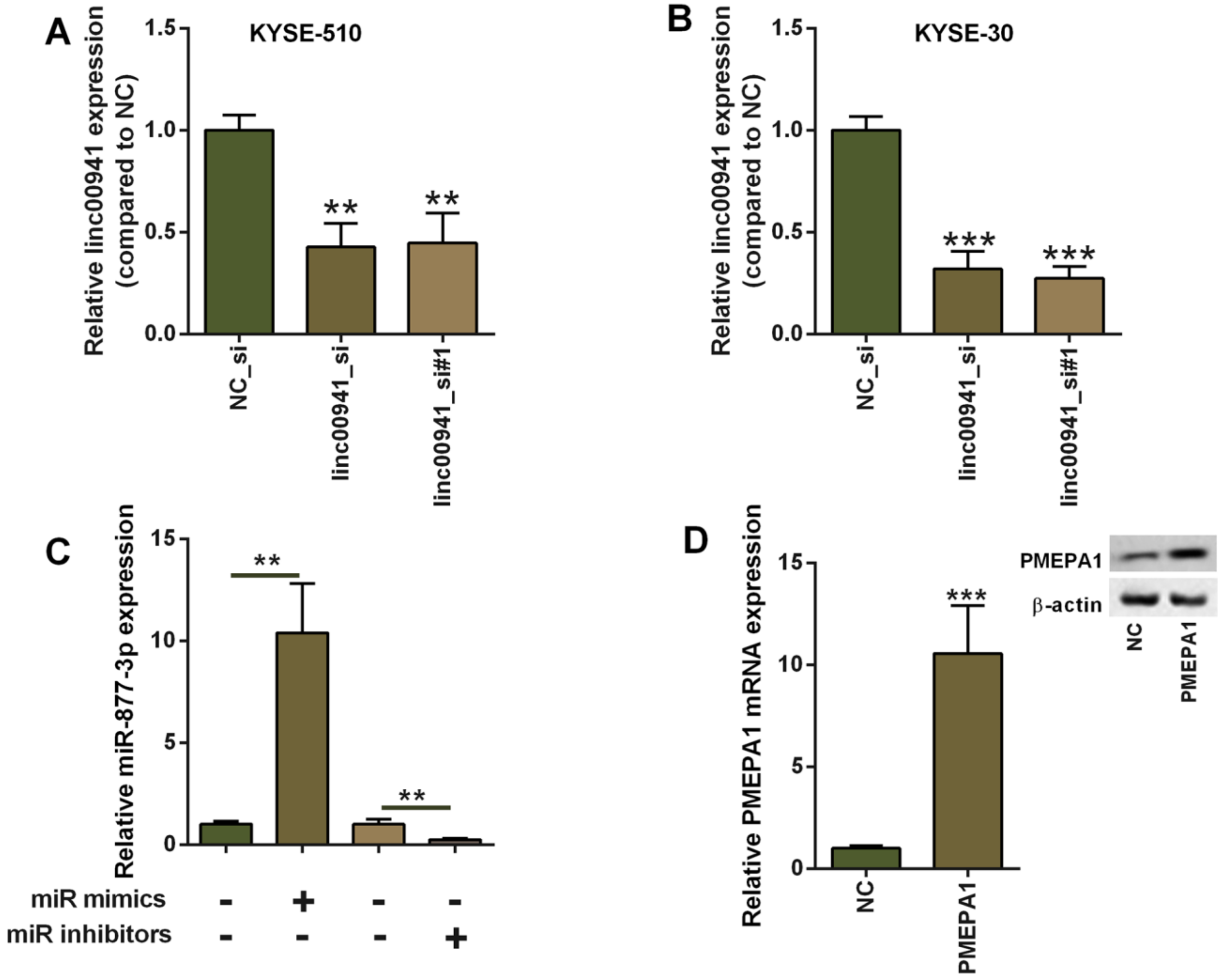

Supplementary Figure 1. Expression of linc00941 in KYSE-510 (**A**) and KYSE-30 (**B**) cells after linc00941 siRNAs or scrambled siRNA transfection. (**C**) Expression of miR-877-3p in KYSE-510 cells after miR-877-3p mimics, inhibitors, mimics NC or inhibitors NC transfection. (**D**) Expression of PMEPA1 mRNA protein levels in KYSE-510 cells after pcDNA3.1 or pcDNA3.1-PMEPA1 transfection.

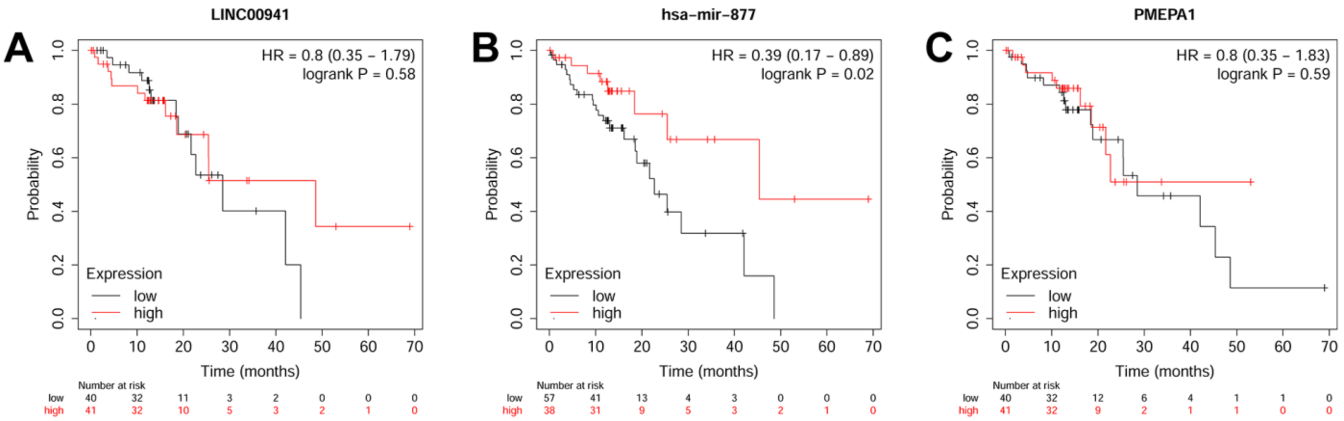

Supplementary Figure 2. Survival analysis of linc00941, miR-877-3p and PMEPA1 in ESCC. The association between linc00941 (**A**), miR-877-3p (**B**) and PMEPA1 (**C**) and overall survival analysis of patients with ESCC.
